# Supplementary material for: Content-rich biological network constructed by mining PubMed abstracts
Source: BMC Bioinformatics. 2004 Oct 8;5:147. doi: 10.1186/1471-2105-5-147 (PMC528731; doi:10.1186/1471-2105-5-147)
Supplement: Additional File 2 — The original results of the above study (non-essential files are deleted to keep the file size under the limit set by BMC bioinformatics). [file 1471-2105-5-147-S2.bz2 › chilibotAdditionalFile2/dip10/364ID8663044E6037/html/FGFR2_FGF3.html]

 


 **FGFR2** and **FGF3** 
  
Found 33 abstracts in PubMed, retrieved 10.  
 

 What does Google say? 
 PDF only 
| .edu only 

---

**Interactive relationship** (e.g. stimulation, inhibition, etc)

**Neutral relationship**- In addition, hindbrain derived  **FGF3**  might regulate otocyst morphogenesis through FGFR 2  [ **FGFR2** ]  IIIb.  Ref: 10934262 J Neurosci, 2000
- Keratinocyte growth factor receptor  [ **FGFR2** ]  KGFR  [ **FGFR2** ] , also known as fibroblast growth factor receptor FGFR 2  [ **FGFR2** ]  IIIb, is located in many types of epithelial cells and is activated by four known ligands FGF 1, FGF 3  [ **FGF3** ] , FGF 7 also known as KGF and FGF 10 that are predominantly synthesized by mesenchymal cells.  Ref: 12608893 Pathol Int, 2003

**Non-interactive relationship** (e.g. studied together, co-existance, homology, etc.)

- The truncated  **FGFR2** , the high affinity FGFR for  **FGF3** , is significantly more efficient at reverting the transformed phenotype as the IIIc isoform, reaffirming the notion that the affinity of the ligand to the DN  **FGFR2**  isoform determines the effect.  Ref: 12084721 J Biol Chem, 2002
- In later development,  **FGF3**  or FGF10 acting on FGF receptor  [ **FGFR2** ]  2b is likely to be involved in development of the walls of the cochlear spaces, while FGF receptor  [ **FGFR2** ]  3 is involved in differentiation of the pillar cells of the organ of Corti.  Ref: 11914524 Audiol Neurootol< MedlineTA>Audiol Neurootol, 2002
